# Supplementary material for: The low-density lipoprotein receptor and apolipoprotein E associated with CCHFV particles mediate CCHFV entry into cells
Source: Nat Commun. 2024 May 28;15:4542. doi: 10.1038/s41467-024-48989-5 (PMC11133370; doi:10.1038/s41467-024-48989-5)
Supplement: Supplementary file 1 — Supplementary Information [file 41467_2024_48989_MOESM1_ESM.pdf]

Supplementary Information

The low-density lipoprotein receptor and apolipoprotein E associated with CCHFV particles mediate CCHFV entry into cells

Maureen Ritter, Lola Canus*, Anupriya Gautam*, Thomas Vallet*, Li Zhong*, Alexandre Lalande, Bertrand Boson, Apoorv Gandhi, Sergueï Bodoirat, Julien Burlaud-Gaillard, Natalia Freitas, Philippe Roingeard, John N. Barr, Vincent Lotteau, Vincent Legros, Cyrille Mathieu, François-Loïc Cosset**, and Solène Denolly**

* These authors contributed equally
** These authors jointly supervised this work

Corresponding authors : francois-loic.cosset@ens-lyon.fr; solene.denolly@ens-lyon.fr

Supplementary Table 1. Antibodies used in this study.

Primary antibodies		
**Targets**	**Dilution**	**Company (catalog number)**
LDL-R (goat)	IF 20mg/mL;FACS 40mg/mL ; WB 2mg/mL; blocking assay 0.25-1-4mg/mL	R&D systems (AF2148)
Lrp1 (rabbit)	WB 1:1000	Abcam (EPR3724)
VLDL-R (mouse)	FACS 1:1000	Abcam (1H10)
SR-BI (mouse)	WB 1:200	BD biosciences (610883)
CD81 (mouse)	IF 1:250	BD Pharmingen (JS-81)
apoE (goat)	IP 10µL	Sigma-Aldrich (AB947)
apoE (goat)	WB 1:2000; EM 1:100; neutralization assay 1:200-1:100-1:50; FACS 1:2000	AbD Serotec (AHP2177)
Actin (mouse)	WB 1:10000	Sigma-Aldrich (AC-74)
Calnexin (rabbit)	WB 1:1000	Enzo Life sciences (ADI-SPA-865-F)
CCHFV Gc 11E7 (mouse)	WB 1:1000	BEI Resources (NR-40277)
CCHFV Gn (mouse)	WB 1:1000 ; EM 1:100	Home-made
CCHFV NP 9D5 (mouse)	WB 1:1000	BEI Resources (NR-40277)
CCHFV NP 2B11 (mouse)	Immunostaining 1:250	BEI Resources (NR-40257)
HCV NS5A 9E10 (mouse)	Immunostaining 1:800	Kind gift from C. Rice
Anti VSV-G (mouse)	Neutralization for production 1:100	41A1 (hybridoma)
Goat IgG	FACS 40ug/mL; blocking assay 0.25-1-4ug/mL	ThermosFisher (02- 363 6202)
Goat serum	neutralization assay 1:200-1:100-1:50	Viromed (79S094)
**Secondary antibodies**		
Donkey anti-mouse Alexa Fluor 555	IF 1:2000	ThermoFisher (A-31570)
Donkey anti-goat Alexa Fluor 488	IF 1:2000	ThermoFisher (A-11055)
Goat anti-Mouse-HRP	Immunostaining (HCV) 1:1000	Sigma-Aldrich (A4416)
Anti-Mouse IgG (whole molecule)–Peroxidase antibody	Immunostaining (CCHFV) 1:1000	Sigma-Aldrich (A5278)
Goat F(ab')2 Anti-Mouse Ig, Human ads-APC	FACS 1:100	Southern Biotech (1012-11)
F(ab')2-Donkey anti-Goat IgG (H+L), PE	FACS 1:100	ThermoFisher (31860)
Anti-Goat IgG, FITC	FACS 1:100	Dako (F0250)
IRDye® 680RD Donkey anti-Mouse IgG	WB 1:10000	Li-COR Biosciences (926-68072)
IRDye® 800CW Goat anti-Mouse IgG	WB 1:10000	Li-COR Biosciences (926-32210)
IRDye® 800CW Donkey Anti-Rabbit IgG	WB 1:10000	Li-COR Biosciences (926-32213)
IRDye® 800CW Donkey anti-Goat IgG	WB 1:10000	Li-COR Biosciences (926-32214)

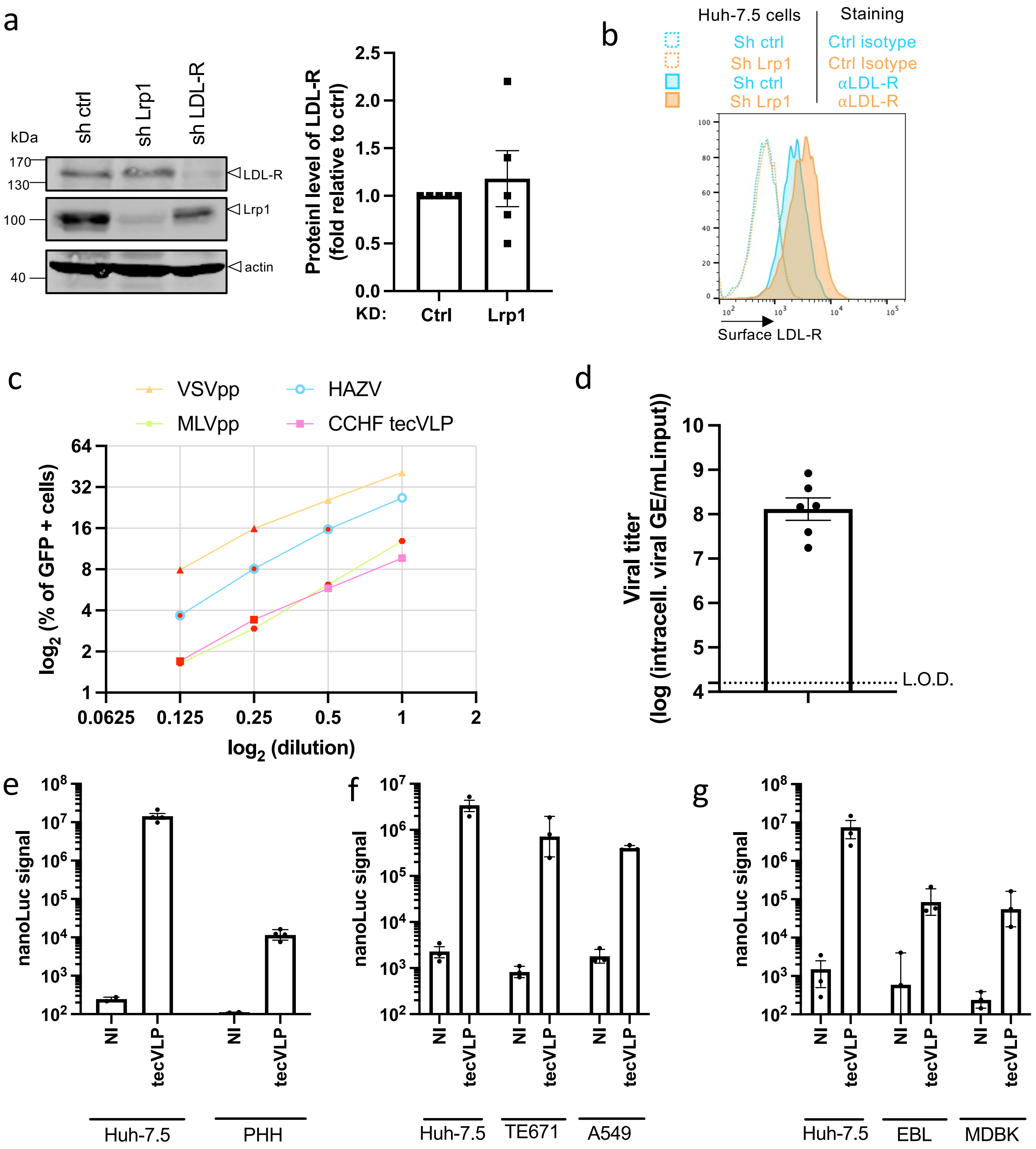

Supplementary Figure 1. Conditions for transduction or infection with tecVLP or WT CCHFV.
(**a**) Western blot analysis of cell lysates from Huh-7.5 cells stably expressing FLuc, transduced with lentiviral vectors allowing expression of control shRNA or shRNA targeting Lrp1 or LDL-R (left). Representative image of 5 independent experiments. Quantification of abundance of LDL-R (right) in control or Lrp1 KD cells. N=5 independent experiments. (**b**) Cell surface expression of LDL-R of cells transduced with sh-ctrl or sh-Lrp1 as described in Figure 1b. Control isotypes are depicted in dotted lines. Representative image of 3 independent experiments (**c**) Representative example of the linearity of infection or transduction obtained with serial dilutions of particles. The red dots indicate the values used for calculation of the levels of infection/transduction. (**d**) Viral titers obtained for experiments with WT CCHFV described in Figure 1a. N=6 independent experiments. (**e**) Levels of nanoLuc signals detected at 24h after transduction of Huh-7.5 cells or PHH with 100μL of tecVLPs with minigenome encoding nanoLuc. N=4 independent experiments. (**f**) Levels of nanoLuc signals detected 48h after transduction of Huh-7.5, TE-671 and A549 cells with 100μL of tecVLPs with minigenome encoding nanoLuc. N=3 independent experiments (**g**) Levels of nanoLuc signals detected 48h after transduction of Huh-7.5, EBL and MBDK cells with 100μL of tecVLPs encoding nanoLuc. N=3 independent experiments. NI=non infected control. Data are represented as the means ± SEM. Each dot in the graphs corresponds to the value of an individual experiment.

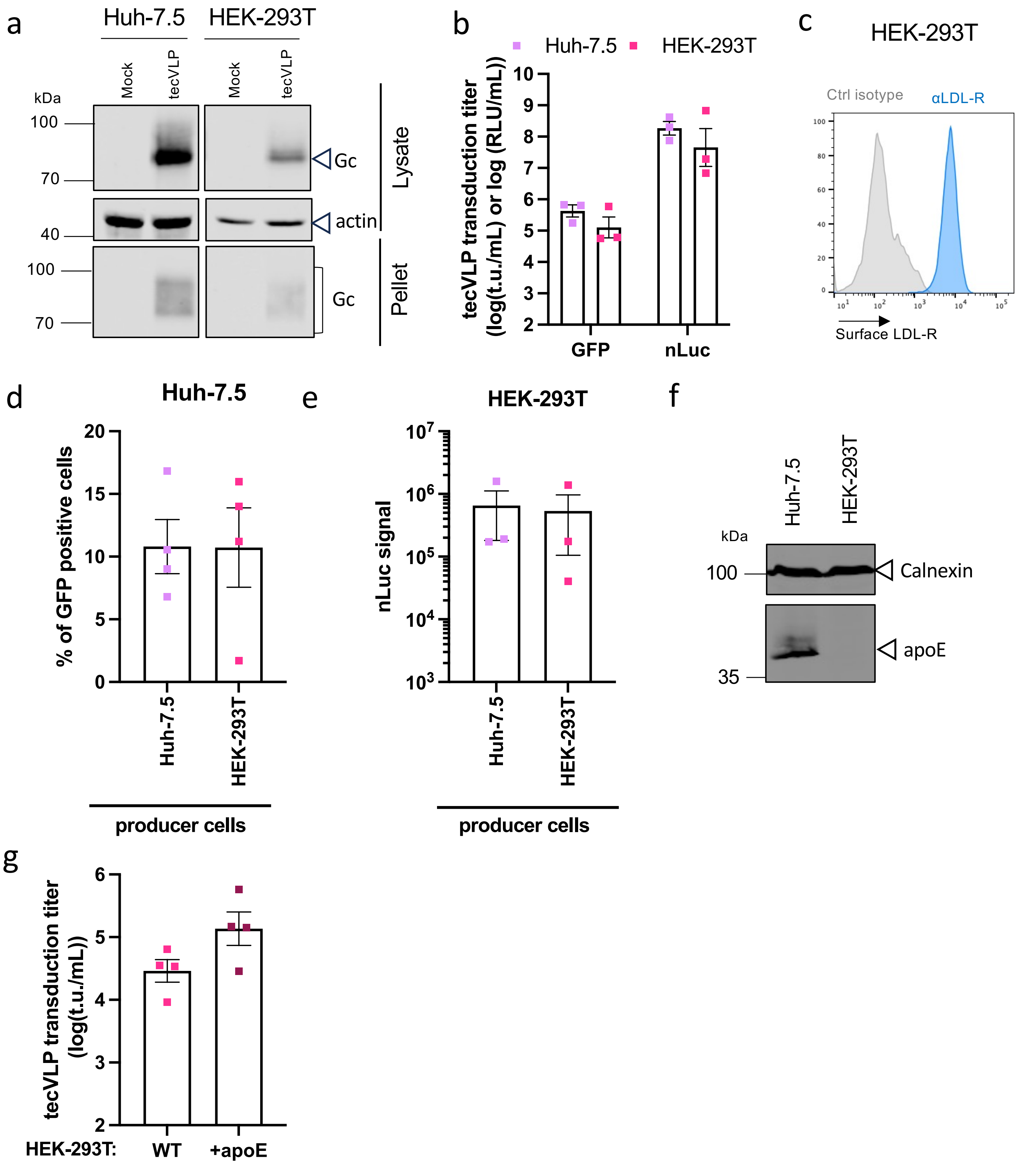

Supplementary Figure 2. apoE influences CCHF tecVLPs infection.

(**a**) Western blot of cell lysates and pellets of tecVLPs produced in Huh-7.5 or HEK-293T cells and revealed for Gc expression. Representative image of 3 independent experiments. (**b**) Transduction titer of tecVLP-GFP or tecVLP-NanoLuc particles produced in Huh-7.5 (violet) or HEK-293T (pink) cells. The infectivity was assessed after transduction of Huh-7.5 cells pre-transfected with NP+L expression plasmids, by flow cytometry at 24h post-transfection (p.t.) for GFP minigenome or after transduction of Huh-7.5 cells, by nanoLuc signal measurement at 24h p.t. for nanoLuc minigenome. N=3 independent experiments. (**c**) Cell surface staining of LDL-R at the surface of HEK-293T cells. Representative images of 3 independent experiments. (**d**) Percentage of GFP positive cells obtained for condition without antibodies in experiments of Figure 3b (left). N=3 independent experiments. (**e**) Levels of nanoLuc signal obtained for condition without antibodies in experiments of Figure 3b (right). N=3 independent experiments. (**f**) Western blot of cell lysates of Huh-7.5 and HEK-293T cells that were revealed for expression of apoE. Representative image of 3 independent experiments. (**g**) Transduction titers of tecVLP-GFP particles produced in HEK-293T cells (pink) or in HEK-293T cells ectopically expressing apoE (maroon). The infectivity was assessed after transduction of Huh-7.5 cells pre-transfected with NP+L expression plasmids, by flow cytometry at 24h p.t. for GFP minigenome. N=4 independent experiments

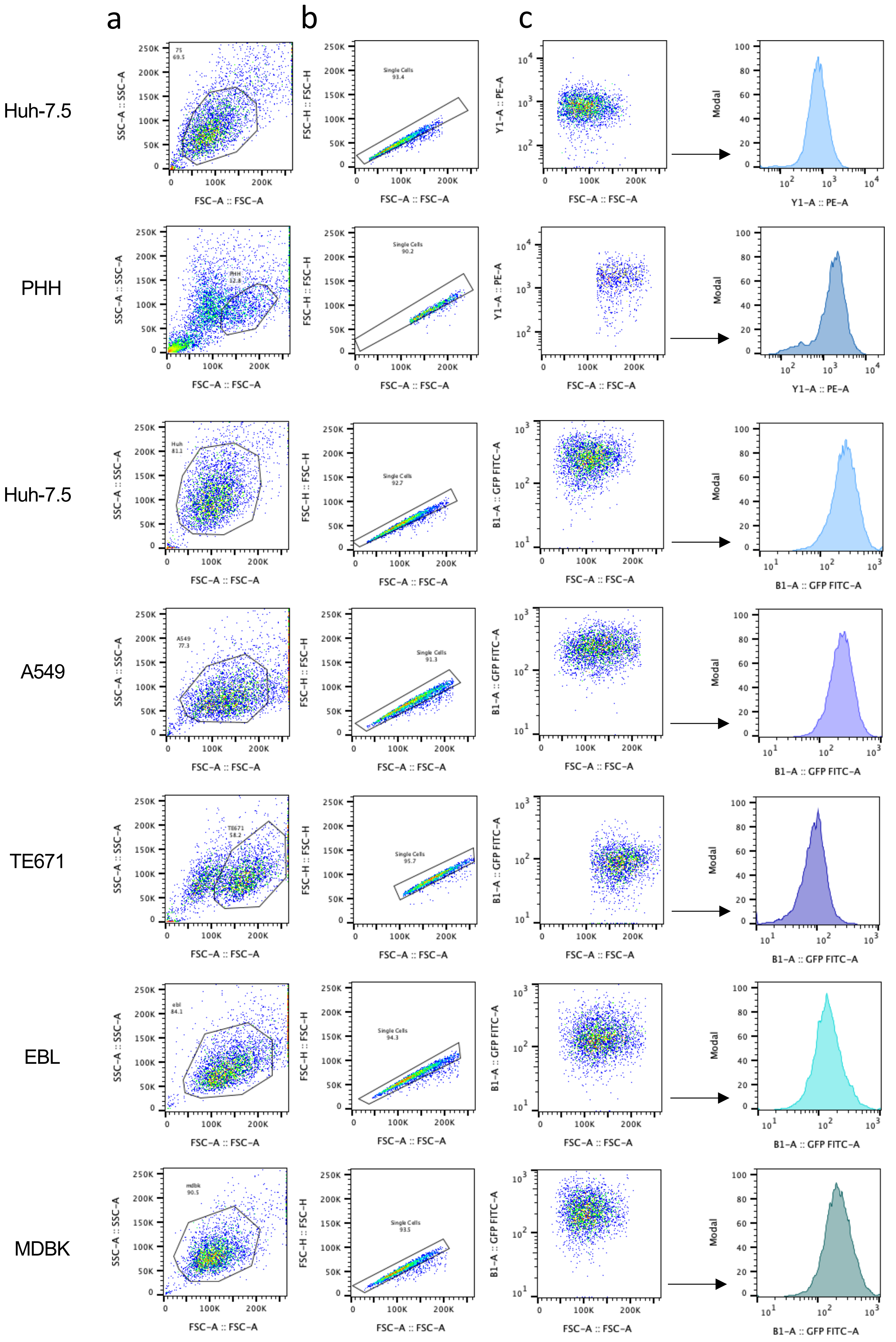

Supplementary Figure 3. Gating strategy for LDL-R cell surface staining.
(**a**) The population of viable cells was first identified by creating a forward *vs*. side scatter plot. (**b**) The population identified in (a) was then then gated to isolate single cells only, by plotting the forward scatter area by height and gating for cells that display a linear relationship. **(c)** Cells from (b) were displayed as a histogram of the fluorescent intensity of the fluorophore of interest. Strategy depicted here refers to Fig. 2a. Same strategy was used for Fig. 7.

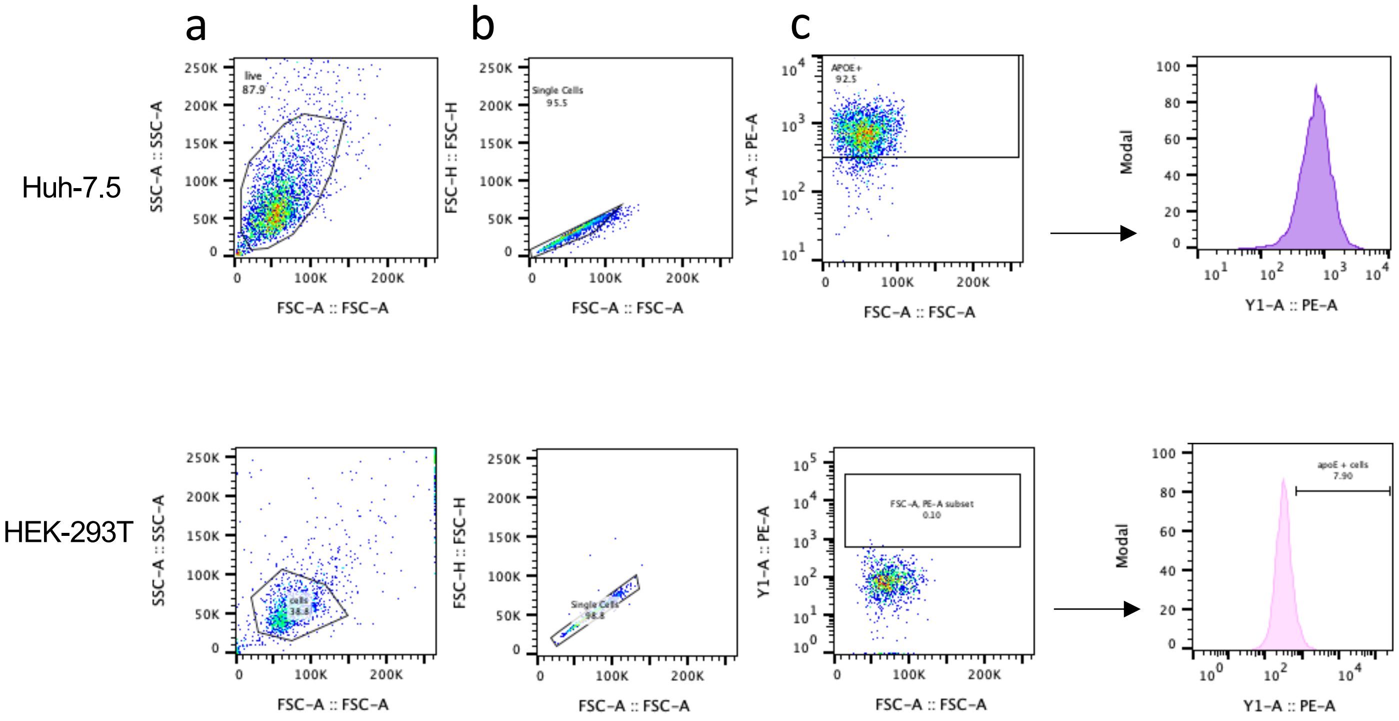

Supplementary Figure 4. Gating strategy for apoE intracellular staining.

(**a**) The population of viable cells was first identified by creating a forward *vs*. side scatter plot. (**b**) The population identified in (a) was then then gated to isolate single cells only, by plotting the forward scatter area by height and gating for cells that display a linear relationship. **(c)** Cells from (b) were displayed as a histogram of the fluorescent intensity of the fluorophore of interest. Strategy depicted here refers to Fig. 4c and Fig. 6e
