# Supplementary material for: The low-density lipoprotein receptor and apolipoprotein E associated with CCHFV particles mediate CCHFV entry into cells
Source: Nat Commun. 2024 May 28;15:4542. doi: 10.1038/s41467-024-48989-5 (PMC11133370; doi:10.1038/s41467-024-48989-5)
Supplement: Supplementary file 3 — Reporting Summary [file 41467_2024_48989_MOESM3_ESM.pdf]

nature portfolio

Corresponding author(s): COSSET François-Loïc

Last updated by author(s): Apr 24, 2024

Reporting Summary

Nature Portfolio wishes to improve the reproducibility of the work that we publish. This form provides structure for consistency and transparency in reporting. For further information on Nature Portfolio policies, see our Editorial Policies and the Editorial Policy Checklist.

Statistics

For all statistical analyses, confirm that the following items are present in the figure legend, table legend, main text, or Methods section.

n/a	Confirmed	
☐	☒	The exact sample size (*n*) for each experimental group/condition, given as a discrete number and unit of measurement
☐	☒	A statement on whether measurements were taken from distinct samples or whether the same sample was measured repeatedly
☐	☒	The statistical test(s) used AND whether they are one- or two-sided<br>*Only common tests should be described solely by name; describe more complex techniques in the Methods section.*
☒	☐	A description of all covariates tested
☐	☒	A description of any assumptions or corrections, such as tests of normality and adjustment for multiple comparisons
☐	☒	A full description of the statistical parameters including central tendency (e.g. means) or other basic estimates (e.g. regression coefficient) AND variation (e.g. standard deviation) or associated estimates of uncertainty (e.g. confidence intervals)
☐	☒	For null hypothesis testing, the test statistic (e.g. *F*, *t*, *r*) with confidence intervals, effect sizes, degrees of freedom and *P* value noted<br>*Give P values as exact values whenever suitable.*
☒	☐	For Bayesian analysis, information on the choice of priors and Markov chain Monte Carlo settings
☒	☐	For hierarchical and complex designs, identification of the appropriate level for tests and full reporting of outcomes
☒	☐	Estimates of effect sizes (e.g. Cohen's *d*, Pearson's *r*), indicating how they were calculated

Our web collection on statistics for biologists contains articles on many of the points above.

Software and code

Policy information about availability of computer code

Data collection	-western blot data were collected using Odyssey infrared imaging system CLx (Li-Cor Biosciences)<br>- electron microscope image data were collected using JEM-1400Plus (Jeol)<br>- fluorescence microscopy data were collected using AxioObserver Z1 or LSM-800 (Zeiss).<br>- qPCR data were acquired using QuantStudio real-time PCR apparatus(ThermoFisher) .<br>-flow cytometry data were acquired using MACSQuant® VYB Flow Cytometer (Miltenyi Biotec).
Data analysis	- Images were analyzed with the FIJI software package.<br>- western blot data were analyzed with ImageStudio (version 5.5.4) (Li-Cor Biosciences).<br>- flow cytometry data were analyzed with FlowJo software version 10 (BD Biosciences).<br>- qPCR data were analyzed with QuantStudio software (ThermoFisher).<br>-GraphPad Prism software (version 10) was used for plotting the results and statistical tests.

For manuscripts utilizing custom algorithms or software that are central to the research but not yet described in published literature, software must be made available to editors and reviewers. We strongly encourage code deposition in a community repository (e.g. GitHub). See the Nature Portfolio guidelines for submitting code & software for further information.

Data

Policy information about availability of data

All manuscripts must include a data availability statement. This statement should provide the following information, where applicable:

- Accession codes, unique identifiers, or web links for publicly available datasets
- A description of any restrictions on data availability
- For clinical datasets or third party data, please ensure that the statement adheres to our policy

The data generated in this study are provided in the Source Data file.

Research involving human participants, their data, or biological material

Policy information about studies with human participants or human data. See also policy information about sex, gender (identity/presentation), and sexual orientation and race, ethnicity and racism.

Reporting on sex and gender	NA
Reporting on race, ethnicity, or other socially relevant groupings	NA
Population characteristics	NA
Recruitment	NA
Ethics oversight	NA

Note that full information on the approval of the study protocol must also be provided in the manuscript.

Field-specific reporting

Please select the one below that is the best fit for your research. If you are not sure, read the appropriate sections before making your selection.

- [x] Life sciences
- [ ] Behavioural & social sciences
- [ ] Ecological, evolutionary & environmental sciences

For a reference copy of the document with all sections, see nature.com/documents/nr-reporting-summary-flat.pdf

Life sciences study design

All studies must disclose on these points even when the disclosure is negative.

Sample size	No sample size calculation was performed. Our experiments involved testing infection in cultured cells where sample size is not relevant.
Data exclusions	We did not exclude any data in any of the experiments performed in cell culture
Replication	A minimum of three biological experiments were performed independently. The exact numbers of biological replicates are indicated in the figure legends. All attempts were successful.
Randomization	Our experiments did not involve testing on a large number of individuals or groups of individuals. As such, randomization of samples is not relevant to our study.
Blinding	No blinding was done. Analyses were conducted in an automated manner using software reducing the occurrence of bias.

Reporting for specific materials, systems and methods

We require information from authors about some types of materials, experimental systems and methods used in many studies. Here, indicate whether each material, system or method listed is relevant to your study. If you are not sure if a list item applies to your research, read the appropriate section before selecting a response.

Materials & experimental systems

n/a	Involved in the study	
☐	☒	Antibodies
☐	☒	Eukaryotic cell lines
☒	☐	Palaeontology and archaeology
☒	☐	Animals and other organisms
☒	☐	Clinical data
☒	☐	Dual use research of concern
☒	☐	Plants

Methods

n/a	Involved in the study	
☒	☐	ChIP-seq
☐	☒	Flow cytometry
☒	☐	MRI-based neuroimaging

Antibodies

Antibodies used

anti Lrp1 (EPR3724, Abcam) WB 1:1000
anti LDL-R (AF2148, R&D system) IF 20ug/mL;FACS 40ug/mL ; WB 2ug/mL; blocking assay 0.25-1-4ug/mL
anti apoE (AHP2177; AbD Serotec) WB 1:2000; EM 1:100; neutralization assay 1:200-1:100-1:50
goat serum (79S094;Viromed) neutralization assay 1:200-1:100-1:50
anti SR-B1 (610883; BD Biosciences) WB 1:200
anti VLDL-R (1H10; Abcam) FACS 1:1000
goat IgG (02- 363 6202; ThermoFisher) FACS 40ug/mL; blocking assay 0.25-1-4ug/mL
anti apoE (AB947; Sigma-Aldrich) IP: 10uL
anti actin (AC-74, Sigmal aldrich) WB 1:10000
anti calnexin(ADI-SPA-865-F, Enzo life biosciences) WB 1:1000
anti CCHFV Gc 11E7 (NR-40277; Bei resources)WB 1:1000
anti CCHFV NP 9D5 (NR-40270; Bei resources) WB 1:1000
anti CCHFV NP 2B11 (NR-40257;Bei resources) immunostaining 1:250
anti CCHFV Gn (home-made) WB 1:1000; EM 1:100
anti HCV NS5A antibody (clone 9E10) was a kind gift from Charle Rice immunostaining 1:800
anti CD81 (BD Pharmingen; JS81) IF 1:250
anti VSV-G (hybridoma, 41A1) neutralization 1:100

donkey anti-mouse Alexa Fluor 555 (A-31570) IF 1:2000
donkey anti-goat Alexa Fluor 488 (A-11055) IF 1:2000
goat anti mouse HRP (Sigma-Aldrich A4416) Immunostaining 1:1000
anti mouse IgG-peroxidase Ab (Sigma-Aldrich A5278) Immunostaining 1:1000
Goat F(ab')2 Anti-Mouse Ig, Human ads-APC (Southern Biotech, 1012-11) FACS 1:100
F(ab')2-Donkey anti-Goat IgG (H+L), PE (ThermoFisher ,31860) FACS 1:100
Anti-Goat IgG, FITC (Dako (F0250)) FACS 1:100
IRDye® 680RD Donkey anti-Mouse IgG (Li-COR Biosciences (926-68072)) WB 1:10000
IRDye® 800CW Goat anti-Mouse IgG (Li-COR Biosciences (926-32210)) WB 1:10000
IRDye® 800CW Donkey Anti-Rabbit IgG (Li-COR Biosciences (926-32213)) WB 1:10000
IRDye® 800CW Donkey anti-Goat IgG (Li-COR Biosciences (926-32213)) WB 1:10000

Validation

antibodies targeting CCHFV proteins were validated in previous articles (Freitas et al. 2020) or were validated by running Mock samples in parallel.
anti NS5A was validated in Boson et al., 2020
commercial antibodies targeting apoE, SR-BI, VLDL-R, Lrp1, LDL-R, CD81 were validated by the manufacturers and used according to their recommendations. We further validate them in our hand by analyzing samples knock-down or negative for the protein of of interest. The data are provided in the manuscript for apoE and LDL-R. Commercial antibodies targeting actin and calnexin were validated by the manufacturers.

Eukaryotic cell lines

Policy information about cell lines and Sex and Gender in Research

Cell line source(s)

Huh-7.5 cells (male) were a kind gift from Charles Rice
A549 cells (male) were kind gift from P. Boulanger
HEK-293T kidney cells (female) were purchased at ATCC (CRL-3216)
TE671 cells (female) were purchased at ATCC (CRL-8805)
VeroE6 cells (female) were purchased at ATCC (CRL-1586)
EBL cells (sex not known) were kind gift from F. Larcher
MDBK cells (male) were obtained form European Collection of Authenticated Cell Cultures (ECACC)
Primary human hepatocytes were purchased at BD Biosciences

Authentication

no authentification was done

Mycoplasma contamination	all the cell lines are negative to mycoplasma (test performed monthly).
Commonly misidentified lines (See ICLAC register)	None of the cells are in the list

Plants

Seed stocks	NA
Novel plant genotypes	NA
Authentication	NA

Flow Cytometry

Plots

Confirm that:

- [x] The axis labels state the marker and fluorochrome used (e.g. CD4-FITC).
- [x] The axis scales are clearly visible. Include numbers along axes only for bottom left plot of group (a 'group' is an analysis of identical markers).
- [x] All plots are contour plots with outliers or pseudocolor plots.
- [x] A numerical value for number of cells or percentage (with statistics) is provided.

Methodology

Sample preparation	Staining were done on fixed cell lines (fixation with 2-4% PFA)
Instrument	Macsquant VYB
Software	FlowJo (version 10)
Cell population abundance	10 000 cells were acquired in live cells (FSC-SSC gating)
Gating strategy	1) gating in FSC-A/SSC to select live cells<br>2) gating in FCS-A/FSC-H to select single cells<br>3)a- for titration, FCS-A/GFP/FITC was used to select infected cells (Gating was made with mock cells)<br>b- for cell surface staining of LDL-R (or VLDLR) , histograms showing the relative count vs. LDL-R (VLDLR) staining were plotted. Control IgG were used in control.<br>c- for intracellular staining of apoE, histograms showing the relative count vs. apoE staining were plotted.Control IgG were used in control.

- [x] Tick this box to confirm that a figure exemplifying the gating strategy is provided in the Supplementary Information.
